# Analysis of Polymorphisms and Haplotype Structure of the Human Thymidylate Synthase Genetic Region: A Tool for Pharmacogenetic Studies

**DOI:** 10.1371/journal.pone.0034426

**Published:** 2012-04-05

**Authors:** Soma Ghosh, M. Zulfiquer Hossain, Michael Borges, Michael G. Goggins, Roxann G. Ingersoll, James R. Eshleman, Alison P. Klein, Scott E. Kern

**Affiliations:** 1 Department of Oncology, Johns Hopkins School of Medicine, Johns Hopkins University, Baltimore, Maryland, United States of America; 2 Department of Pathology, Sidney Kimmel Comprehensive Cancer Center, Johns Hopkins School of Medicine, Johns Hopkins University, Baltimore, Maryland, United States of America; 3 Johns Hopkins School of Medicine, Johns Hopkins University, Baltimore, Maryland, United States of America; The Chinese University of Hong Kong, Hong Kong

## Abstract

5-fluorouracil (5FU), a widely used chemotherapeutic drug, inhibits the DNA replicative enzyme, thymidylate synthase (Tyms). Prior studies implicated a VNTR (variable numbers of tandem repeats) polymorphism in the 5′-untranslated region (5′-UTR) of the *TYMS* gene as a determinant of Tyms expression in tumors and normal tissues and proposed that these VNTR genotypes could help decide fluoropyrimidine dosing. Clinical associations between 5FU-related toxicity and the *TYMS* VNTR were reported, however, results were inconsistent, suggesting that additional genetic variation in the *TYMS* gene might influence Tyms expression. We thus conducted a detailed genetic analysis of this region, defining new polymorphisms in this gene including mononucleotide (poly A:T) repeats and novel single nucleotide polymorphisms (SNPs) flanking the VNTR in the *TYMS* genetic region. Our haplotype analysis of this region used data from both established and novel genetic variants and found nine SNP haplotypes accounting for more than 90% of the studied population. We observed non-exclusive relationships between the VNTR and adjacent SNP haplotypes, such that each type of VNTR commonly occurred on several haplotype backgrounds. Our results confirmed the expectation that the VNTR alleles exhibit homoplasy and lack the common ancestry required for a reliable marker of a linked adjacent locus that might govern toxicity. We propose that it may be necessary in a clinical trial to assay multiple types of genetic polymorphisms in the *TYMS* region to meaningfully model linkage of genetic markers to 5FU-related toxicity. The presence of multiple long (up to 26 nt), polymorphic monothymidine repeats in the promoter region of the sole human thymidylate synthetic enzyme is intriguing.

## Introduction

5-Fluorouracil (5FU) was developed by Heidelberger and colleagues [1] as the first generation of chemotherapeutic agent active for gastric cancer. Over time, this drug had been widely used to treat malignancies of the breast, head and neck, and other solid tumors in cancer patients. 5FU causes cell death by incorporating fluorinated nucleotides into DNA and RNA, by covalent binding of its metabolites with Tyms protein, and by inhibiting cell growth through disruption of rRNA processing by the exosome complex [2], [3], [4], [5], [6]. An orally administered analog, capecitabine, is converted metabolically to 5FU.

5FU dosing is typically based on the body surface area of the patient; however, it has not been well standardized Yet, this practice is associated with high amount of variability in plasma 5FU levels, up to 100-fold [7], leading to undesired side-effects. This interpatient and intrapatient variability may be a major contributor to toxicity and subsequent treatment failure [8], [9]. Dose management of 5FU could therefore prove essential to reducing 5FU toxicity in patients.

Multiple variables might affect 5FU therapy. *TYMS* genetic polymorphisms in the 5′- and 3′-UTR have been studied for decades and proposed to influence Tyms protein levels. The 28 bp variable number of tandem repeats (VNTR) in the *TYMS* 5′UTR has been studied extensively. Although up to nine repeats have been observed, the double repeat (2R) and the triple repeat (3R) are far more prevalent [10], [11]. A single G/C nucleotide polymorphism in the 3R sequence gives rise to a 3Rc or a 3Rg triple repeat structure [11]. A 6-bp insertion/deletion polymorphism in the 3′-UTR of the gene is also described [12]. Prior studies supported these 5′UTR repeats as important determinants of Tyms expression in tumors and normal tissues and proposed that the *TYMS* VNTR genotypes could be used to help decide fluoropyrimidine dosage in patients [13], [14], [15]. This proposal however lacked a strong ‘*in-vitro*’ experimental basis (see refutation in [16]).

Several clinical trials were conducted in the light of the above proposal using germline genotype data, which can differ significantly from the aneuploid tumor's genotype. These studies proposed that the germline genotype of the 5′UTR of the *TYMS* gene predicted 5FU-related toxicity [17], [18], [19], [20] while another study concluded that none of the *TYMS* UTR polymorphisms could explain 5FU-related toxicity or clinical outcome [21]. Some studies suggested that patients with the 2R/2R genotype had a higher likelihood of suffering from grade 3–4 toxicity in response to 5FU-based therapy as compared to patients with 3R/3R genotype [19]; the results of these studies were largely inconsistent and presented a broad range of odds ratios. A recent Blue Cross Blue Shield (BCBS) opinion (BCBS report, Volume 24, No. 13, August, 2010) refuted these toxicity studies and concluded that the determination of the VNTR alone could not reliably predict toxicity from 5FU.

We therefore wanted to reexamine this subject. We suspected that the literature could be reconciled were there an extended genetic haplotype in the *TYMS* 5′UTR, perhaps predictive of 5FU toxicity. The mononucleotide repeats and SNPs in this region have received little published attention. It would be important to characterize these additional polymorphisms in order to define a ‘complete’ genotype of an individual. To better understand these findings we first conducted a detailed genetic analysis of the *TYMS* locus. Through detailed sequence analysis, we report three types of polymorphisms in this region: the known VNTRs, mononucleotide repeats of the promoter region, and the SNPs of the wider genomic context. These polymorphisms when taken together, but not in isolation, define the genotype of an individual, and knowledge of linkage disequilibrium among them might provide clues to understand variation in 5FU-related toxicity in patients.

## Results

### Characterizing three polymorphic forms in the *TYMS* 5′UTR by sequencing

The VNTR types, namely 2R, 3Rc, and 3Rg, were ascertained for both alleles from constitutional DNA in 40 pancreatic cancer patients (Table 1). Of these 40 patients, 31 were Caucasian and their VNTR types were included in our haplotype analysis (details below); linkage disequilibrium structure varies between different populations, and haplotype analysis is known to be population-specific. The twelfth nucleotide in each 28-bp VNTR is a G or a C such that the wild-type sequence is 2RGC (2R, in this study) for the two-repeat VNTR, and 3RGGC (3Rg, in this study), for the three-repeat VNTR. We observed the 3RGCC polymorphism in several individuals (3Rc, in this study) and include this polymorphism in our analysis. We, however, failed to identify 2RCC, 2RGG, and 3RCCC polymorphisms (rare polymorphisms reported [22], [23]) in the 80 chromosomes genotyped in this study and in another 100 chromosome genotyped earlier (data not shown) and therefore do not include these rare polymorphisms in our haplotype analysis.

**Table 1:** VNTR genotypes among 40 genomes.

Genotype	Count
2R/2R	10
2R/3Rc	10
2R/3Rg	7
3Rc/3Rg	6
3Rg/3Rg	4
3Rc/3Rg	3

**Figure 1:**
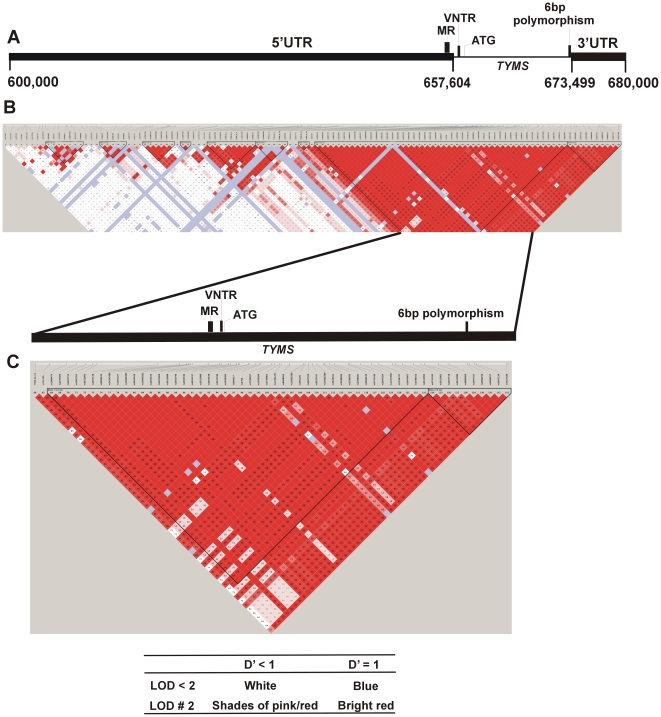
Haplotype structure of the *TYMS* genetic region. **A.** Linear map of the 80 kb *TYMS* genetic region covering the *TYMS* gene (coordinates 657,604–673,499), upstream region (600,000–657,603) and downstream region (673,500–680,000). All coordinate positions are according to UCSC genomic build GRCh37/hg19. SNPs along this region were selected from the HapMap database (see Materials and Methods) and from our sequence analysis. **B.** Nine haplotype blocks (in triangular shape), numbered 1 to 9, were obtained by haplotype analysis using Haploview (see Materials and Methods). The reference SNP numbers (rs) are indicated on top. The linkage disequilibrium (D′) is indicated in the small boxes colored red or blue (a color legend is provided). Some newly discovered SNPs that were not in the public database at the time of analysis were named as TYMS_SG 1, 2, 3, 16,19, 22, and 24. At the time of submission of the new SNPs, we noticed they were deposited by others and had assigned SNP numbers of rs12964837, rs11872762, rs11877806, rs36124867, rs75363899, rs2853533, and rs72634355 respectively. **C.** The largest haplotype block spanning the *TYMS* gene and some parts in the 5′ UTR, including the VNTR and the mononucleotide repeats, and the 3′UTR, is expanded. Blocks 1 and 2 in this figure corresponds to blocks 8 and 9 respectively, of Figure 1B. The unmatched marker 87 corresponding to SNP number rs3826626 (in panel B) was removed in this figure. The locations of the VNTR, MR (mononucleotide repeats), and the 6-bp deletion/insertion polymorphism are shown. The *TYMS* translational start codon is 13 bp downstream of the VNTR. Enlarged versions of figures B and C are provided in supporting information as figure S2 and figure S3, respectively.

We identified some SNPs, not reported hitherto in the public databases, by sequencing 15 kb upstream of the *TYMS* ATG (Figure 1A), using primer pairs spanning 400–500 bp each. We noted 25 SNPs in this 15 kb region, of which 22 are currently in public databases. The remaining three novel SNPs (included in Table S1) were infrequent and were not contributory to our analysis.

Several arrays of mononucleotide repeats in the *TYMS* promoter region were encountered. Three stretches of A's (or T's) were located about 750 bp upstream of the ATG and an additional three stretches were located another 1.5 kb upstream (Figure S1). The existence of the mononucleotide repeats made these regions extremely difficult to sequence due to polymerase stutter. In order to resolve the lengths, we attached a 5′FAM (5-carboxyfluorescein) molecule to the forward primers and used capillary electrophoresis [(CE), ABI 3130] assay to estimate the number of bases. We analyzed these repeat stretches in 32 individuals and found polymorphism in some repeats, discussed in detail below. Repeat polymorphism was confirmed by mixing templates of individuals exhibiting differing repeat lengths, followed by the CE assay (Tables 2, 3).

**Table 2:** Polymorphism among mononucleotide repeats near the *TYMS* promoter.

Mononucleotide repeat	No. of samples examined	Position in genome^1^	Polymorphism observed
MR1 (A_15_GA_9_)	32	655,156–655,180	None
MR2 (A_24_)	32	655,323–655,346	2–5 bp variation
MR3 (T_14_C_4_)	32	656, 030–656,047	2–3 bp variation
MR4 (A_11_)	32	656,712–656,722	None
MR5 (A_16_)	20	656,871–656,886	None
MR6 (T_26_)	22	656,933–656,958	2–3 bp variation

1 UCSC genomic build GRCh37/hg19.

**Table 3:** Length variability (polymorphism) within the MR2 repeat.

Sample	Peak location	Peak height^1^	Peak width^2^
PN8	245	1435	2
PN9	250	1572	3
PN33	249	3963	2
PN38	245	1176	3
PN79	245	1206	3
PN104	245	2904	2
PN9+PN104 (1∶2)	247	1754	4–5^3^

1 Machine-generated measurement of DNA length at the top of the observed peak, in nucleotide scale.

2 Peak width at half-maximal heights of both peak slopes, in nucleotide scale.

3 The peak width was between 4 and 5 nucleotides (half-maximal peak height for this sample was more than 4 nucleotides but less than 5). Analysis of the graphs from mixtures made of differing ratios produced a migration of the peak as expected, confirming a variation in the polymorphic forms of samples PN9 and PN104.

### Length analysis of mononucleotide repeats

Mononucleotide repeats could be useful as markers for genetic mapping. In addition to being abundant, they have considerable length variability or polymorphism. The presence of six stretches of mononucleotide repeats (MR) (Figure S1; Table 2), particularly poly (A) and poly (T), within 2 kb upstream of the *TYMS* promoter region (Figure S1), was unusual. Among the 32 individuals studied for repeat lengths, repeats MR2, MR3, and MR6 had length polymorphism (Table 2), ranging between 1- to 5-bp variation. Additional variation could have evaded our study, due to polymerase stutter and due to difficulty in resolving the closely located MR5 and MR6 repeats (Figure S1). A 1-bp difference was considered as within the variation of the instrument [standard deviation of 0.04 to 0.24 nucleotides, [24]] but greater differences were considered a polymorphism of repeat lengths. As shown in Table 3, MR2 locus had the most variation (2–5 bp) from among the six repeats analyzed. Using “peak width” measured at the half-maximal points of a peak in each sample, we created mixtures of samples having variation in DNA lengths; in this manner, we further confirmed that the repeat was indeed polymorphic. Specifically, samples PN9 and PN104 (having a 3-bp difference) were mixed in different ratios. The results revealed a broader peak in the mixed samples as compared with the individual samples (depicted in Table 3). The graphs of the primary data produced by the mixed samples had a skewed appearance, as expected, representing the predominant sample in the mixture (data not shown) when the degree of mixture was modified (Table 3).

### SNP genotyping and haplotype structure of *TYMS* genetic region

**Figure 2:**
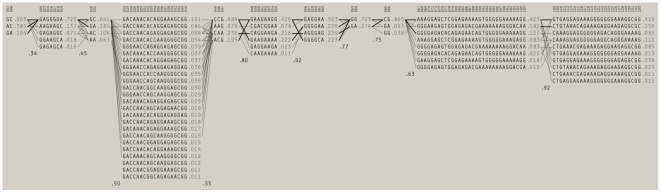
The nine most common SNP haplotypes. Common haplotypes and estimated haplotype frequencies as determined using Haploview across the region under survey. Numbers on top of the figure indicate the ‘SNP number’ from the 80 kb analyzed region (refer Figure 1A) as listed in Table S2. Numbers in the middle reflect frequencies of the individual haplotype. These frequencies sum up to the numbers at the bottom because they reflect only fairly common haplotypes (i.e., the number at the end ‘0.94’, explains frequencies of 94% of individuals, the rest of the individuals have rare haplotypes).

We examined an 80 kb stretch from the *TYMS* genetic region (Figure 1A) to infer the haplotype structure from the SNPs in the region and to determine whether the SNPs were in linkage disequilibrium (D′, a numerical representation of correlation with other forms of polymorphism [25]). We chose 133 SNPs (Table S2) from HapMap and our sequencing data (Table S1), and we studied their distribution in our 351 individuals. Several haplotype blocks numbered 1 to 9 (Figure 1B) were found in this region. The boxes in red or pink (Figure 1B) depict D′ to have very good dependency, or correlation, between SNPs, as shown in the color legend in Figure 1. The boxes in blue or white indicate D′ to have poor dependency, or correlation, between SNPs. D′ is indicated in each box and can be visualized most clearly in Figure S2. The largest haplotype block spanned the *TYMS* gene and some parts upstream, including the VNTR and the mononucleotide repeats (Figure 1C; enlarged version in Figure S3), indicating that the SNPs in the region were very strongly associated with each other. The nine common SNP haplotypes identified in this block (Figure 2) accounted for nearly 92% of the variation among the tested population. The most common haplotype was found on 42% of chromosomes.

### Relationship between SNP haplotypes and VNTR

**Figure 3:**
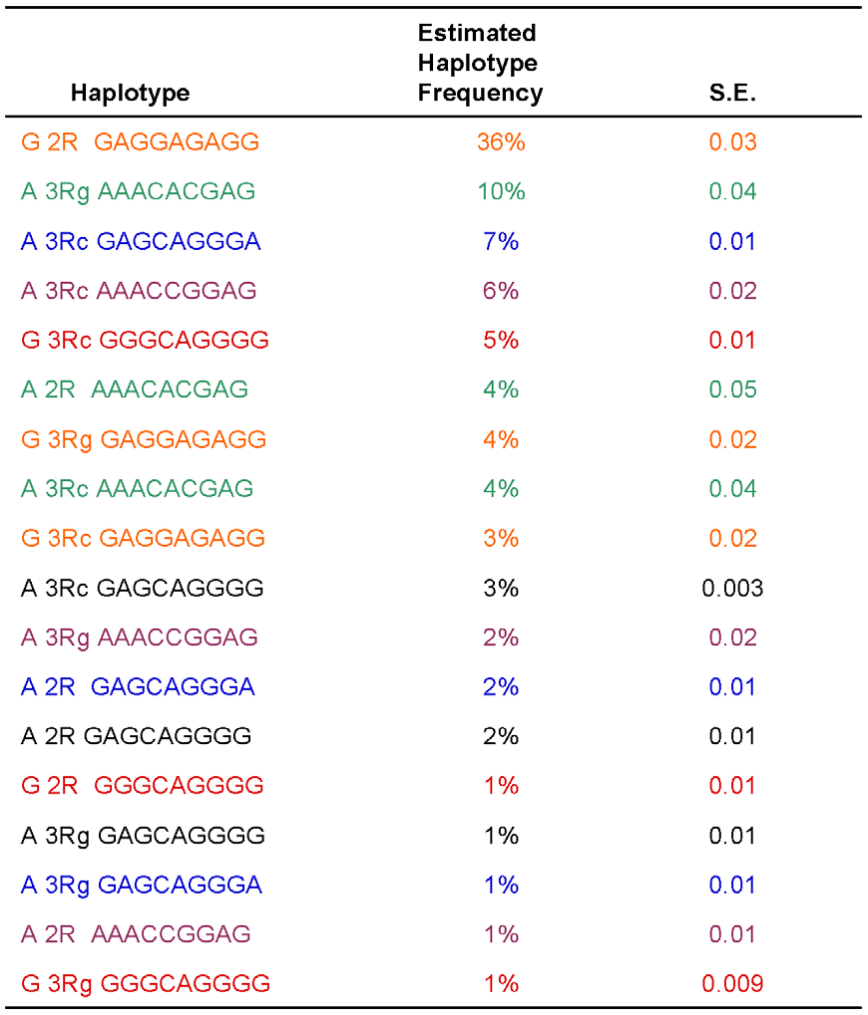
Estimated haplotype frequencies for common haplotype blocks (≥1%) containing a given VNTR type. Haplotypes that included the VNTR were estimated in 322 Caucasian patients using the following marker alleles: rs1001761, VNTR, rs699517, rs3744962, rs9948583, rs495139, rs2298582, rs2298581, rs2471186, rs7236747, and rs2612092. VNTR data was available for 31 patients. Each color represents one SNP-defined haplotype, for SNPs located in blocks 7 and 8 of Figure 1B. The observed frequency (in 31 individuals, 62 chromosomes) for each VNTR is as follows: 2R (50%), 3Rc (32%), and 3Rg (18%). The estimated haplotype frequency of association of the indicated VNTR/haplotype pair is shown and the standard error (SE) is indicated.

The correlation between these haplotypes and the VNTR was weak (depicted in Figure 3). While the frequency of the VNTR alleles varied by haplotype, the same VNTR allele occurred on different haplotype backgrounds at varying frequencies (Figure 3). The VNTR type ‘2R’ was present in the most-common haplotype (36% of alleles); the VNTR type ‘3Rg’ was present in the second-most-common haplotype (10% of alleles), and so on. Our analysis indicated that the 5′UTR VNTR appears to exhibit homoplasy (mechanism of inheritance by convergence, parallelism or reversals and not by common ancestry) [26], [27], [28] and is more rapidly evolving than the SNP haplotypes, as discussed below. Due to the homoplasious nature of the *TYMS* VNTR, we limited the *TYMS* 5′UTR genotyping to 40 individuals. Determination of the VNTR type in additional individuals would not have altered our conclusion about the weak association of the VNTR and SNP haplotypes.

### Relationship between SNP haplotypes and 6-bp 3′UTR insertion/deletion polymorphism

A 6-bp insertion/deletion polymorphism (TTAAAG) in the 3′-UTR of the thymidylate synthase gene was proposed to influence Tyms expression [29], [30] and 5FU-related toxicity in patients [31] in several studies. This polymorphism occurred in less than 20% of the chromosomes analyzed and was observed on multiple haplotype backgrounds (results not shown).

## Discussion

We define a detailed genetic map of the *TYMS* 5′-genetic region comprising three types of polymorphisms and propose that knowledge of this variation and of the linkage disequilibrium between polymorphisms might provide a means to understand 5FU-related toxicity in patients. Hitherto, several clinical trials were analyzed on the basis of the concept that genotyping the *TYMS* VNTR alone would give an estimate of Tyms protein levels in normal tissues and tumors and therefore help in dosing patients with 5FU. Clinical reports have also related the *TYMS* VNTR to 5FU-related toxicity. The design of these studies, however, omitted a comprehensive examination of the genetic variation in the *TYMS* genetic region.

Minisatellites, or VNTRs, can arise readily due to mispairing of repeats in an array [32], particularly by slipped-strand mispairing during DNA replication, by unequal sister chromatin exchange (USCE) during mitosis or meiosis, or by unequal crossover between homologous chromosomes (interallelic recombination) during meiosis. VNTRs can also mutate by gaining or losing repeats one at a time [33]. There is a high mutation rate at VNTR loci, causing the same mutation to occur independently (in parallel) in different lineages (termed homoplasy or homoplasious alleles), which can be mistaken for homologous alleles (inheritance due to common ancestry) [26], [27], [28]. The *TYMS* VNTR variants do not conform to the patterns expected from common ancestry and are therefore concluded to be homoplasious, evolving rapidly, in contrast to the SNPs in the same region. Due to homoplasy, the SNP haplotypes and VNTR types both would need to be determined to define precise *TYMS* genotypes.

Our genotyping data therefore reveal a weak association between SNP haplotypes and the VNTR types. We observe the different VNTR types occurring on any given SNP haplotype (Figure 3), indicating that the VNTR evolved at a faster rate than the SNP haplotype. The most common VNTR, 2R, occurred in a common haplotype of our hospital-based population. Previous clinical literature reported the 2R/2R genotype to be associated with high levels of 5FU toxicity [19]. The inconsistent results of those studies [17], [18], [19] suggested that the 2R repeat itself might not cause differences in Tyms expression, but might be linked genetically to the causative genetic variation in some populations. If the 2R/2R genotype arose due to homoplasy and not due to common ancestry, an apparently homozygous patient in actuality need not have two copies of the same ancestral allele. Therefore, knowledge of both VNTR and surrounding genetic polymorphisms of an individual may be required to define a genetic haplotype that can be associated with a phenotype. For example, a clinical trial could be designed to examine the association between the extended genetic haplotypes in this region and the undesired occurrence of 5FU toxicity.

Mononucleotide repeats occur throughout the human genome. They often harbor mutations [34] and polymorphism [35], providing useful markers for genetic mapping. The presence of six arrays of poly(A) or poly(T) in the *TYMS* promoter region makes the genetic structure of *TYMS* 5′-UTR interesting, aside from their potential as markers. The polymorphic repeats might conceivably affect *TYMS* promoter function directly. Also, upon 5FU administration, fluorouridine misincorporation in DNA might be concentrated in these tracts, perhaps amplifying deoxythymidylate depletion if local repair might interfere with promoter function [36], [37]. This is speculative, but suggests a number of questions to be addressed in future work.

A direct effect of the VNTR on drug response is not supported by our findings. Our studies indicate that the VNTR is homoplasious and therefore should not be uniformly associated with a putative linked locus associated with risk of toxicity. Genotyping in the *TYMS* region should consider the surrounding polymorphisms, because it is possible that a robust predictive marker could emerge from one of the stable changes (like SNPs) assayed here, because the VNTR was inconsistently linked empirically to 5FU toxicity, and because the VNTR is not likely causative of the toxicity [17], [18], [19]. In the literature, when ‘moving’ from one genetically related population (where the association or linkage may be high) to another population (where the linkage may be weak), the VNTR would lose its linkage as a marker for toxicity. In our study therefore, we looked into other polymorphisms in the *TYMS* 5′UTR region with the idea of finding stable mutations not subject to homoplasy, which could serve as markers having greater stability for comparing less-related populations.

A broader perspective also remains essential. Showalter et al [38] analyzed prior studies related to Tyms expression levels and response to 5FU chemotherapy. Little difference was found among response rates between tumors having low- and high-Tyms expression upon analyzing the grouped data. Although admittedly some articles had used greater sophistication in Tyms protein determinations, the authors expressed doubt that a compelling clinical utility had been found in these reports. A recently conducted pilot study concluded that *TYMS* expression and genotyping based on the 5′UTR repeats have no significant impact on the clinical outcome of cancer patients treated with 5FU [39]. Several other variables may affect Tyms expression and 5FU therapy, other than the proposed *TYMS* genetic polymorphisms. Patients with deficiency in dihydropyrimidine dehydrogenase, the rate-limiting enzyme of pyrimidine catabolism in the 5FU metabolic pathway to the inactive 5-fluoro-5, 6-dihydrouracil, suffer from severe 5FU toxicity [40]. Variable 5FU toxicity might relate to variation in methylene tetrahydrofolate reductase, which forms the reduced folate cofactor essential for inhibiting Tyms [20]. Orotate phosphoribosyl-transferase (Oprt), the enzyme necessary for stabilization and formation of the ternary complex [41] following 5FU treatment is also a potential predictor of 5FU effects [42]. The G213A polymorphism in *OPRT* is reported to be associated with grade 3–4 toxicity in response to 5FU therapy [19]. In addition to Tyms levels or the *TYMS* genotypes, there could be a combination of enzymes in the 5FU metabolic pathway or even deficiencies in dietary folate that could affect 5FU response. Investigators studying *TYMS*-5FU interactions will need to consider these and other possibilities.

Based on our results, we suggest that the understanding of *TYMS* repeats to guide 5FU therapy should be updated. In hopes of determining an empirical basis for predicting 5FU toxicity, VNTR and mononucleotide repeats should be genotyped along with a determination of SNPs in the region to define a complete haplotype.

## Materials and Methods

### Patient samples and IRB approval

Constitutional DNA was extracted from normal frozen tissue of 363 pancreatic cancer patients (proteinase K/phenol: chloroform or by Qiagen DNA tissue kit). Tissues for genetic research were used upon written consent under a protocol approved by our IRB, the Joint Committee on Clinical Investigation of The Johns Hopkins University School of Medicine and The Johns Hopkins Hospital. Demographic information including age, gender and ethnicity was obtained from the medical records.

### Primer design, PCR, and sequence analysis

Primers were synthesized by Integrated DNA Technologies (Sequences are available upon request). Primers for analysis of mononucleotide repeats had a 5′FAM modification in the forward primer for analysis by CE. After PCR using Taq DNA polymerase, products were separated on 1% agarose gel in lithium boric acid buffer (LB®, FasterBetter Media LLC) [43], purified (QIAquick PCR Purification Kit, Qiagen), and analyzed by automated sequencing and by the Sequencher program (Gene Codes).

### Lengths of mononucleotide repeats

Primer pairs flanking each mononucleotide repeat were used to PCR-amplify six fragments of interest (Figure S1). Following PCR, we polished the ends of the amplified products using Klenow (NEB # M0210, per manufacturer's instruction) to eliminate size variation due to varying addition of non-templated adenosines. The polished products were first analyzed on a 1% agarose gel, and then mixed with a size standard and formamide, heat-denatured, and fragments resolved using CE.

### SNPs from public database for haplotype analysis

We selected SNPs (listed in Table S2) from the HapMap database http://hapmap.ncbi.nlm.nih.gov/biomart/martview, (mainly from Caucasians), and a few common SNPs from the African-American population. The SNPs spanned an 80 kb region (Figure 1A) that included 57 kb upstream of the *TYMS* start codon, the entire *TYMS* gene (inclusive of exons and introns, 16. 5 kb), and about 6.5 kb downstream of the *TYMS* stop codon. We reasoned that the 80 kb region would possibly encompass the regulatory elements and polymorphisms responsible for Tyms expression. Novel SNPs identified in our sequence analysis were incorporated into our genotyping panel as described.

### SNP genotyping

A total of 147 SNPs were genotyped (Illumina BeadXpress array). Eleven SNPs were excluded, eight due to call rates of zero and three due to atypical clustering. Of the 133 remaining SNPs (Table S2), the minimum call rate was 99.1%. Of the 363 patient DNA samples, seven were not included in the assay due to low DNA quantity, and five samples failed genotyping. The minimum call rate in the remaining individuals was 99.2%. Overall, genotype data was available for 182 males and 169 females. Of the 351 genotyped, 322 reported Caucasian ancestry, and the remaining 29 belonged to other ethnic groups. Data cleaning was conducted using PLINK [44].

### Haplotype analysis

Haplotype analysis was conducted using Haploview [45] for the Caucasian population. Eighteen SNPs were monomorphic in the Caucasian subset, and eight had a MAF (minor allele frequency) <1%; these SNPs were excluded from haplotype analysis. All remaining SNPs had a HWE (Hardy-Weinberg Equilibrium) p-value >0.001. Blocks were defined using the Gabriel *et al* method [25]. In the Caucasian population, additional haplotype analysis including the VNTR repeats was conducted using PHASE 2.1.1 [46]. To estimate the haplotype frequency in Caucasians for the haplotype containing the VNTR, haplotype-tagging SNPs were selected using the TAGGER program with the aggressive mode (2.3 marker haplotype) and an r^2^ threshold of 0.95 [45]. Using the haplotype-tagging SNPs in the haplotype block containing the VNTR or the 3′ UTR, along with the VNTR or 3′ UTR polymorphisms, haplotype frequencies were estimated using PHASE.

## Supporting Information

Figure S1 **Structure of the** ***TYMS*** **genetic region.** The structure of the TYMS genetic region from coordinates 654,843–657,842 (UCSC genomic build GRCh37/hg19) is shown. The mononucleotide repeats (MR) and VNTR appear as bold underlined letters. The coordinates of the MR are reported in Table 2. The *TYMS* promoter is in italics. The primers used to amplify the MR are listed.
(DOC)

Figure S2 **Enlarged version of** **figure 1B** **(in main document).** Nine haplotype blocks (in triangular shape) were obtained by haplotype analysis using Haploview (see Materials and Methods), covering the 80 kb *TYMS* genetic region (depicted in Figure 1A). The reference SNP numbers (rs) are shown on top. The linkage disequilibrium (D′) is indicated in the small boxes colored red or blue as indicated by the color legend. The boxes in red or pink depict D′ to have very good correlation between SNPs. The boxes in blue or white indicate D′ to have poor correlation. Some newly discovered SNPs that were not in the public database at the time of analysis were named as TYMS_SG 1, 2, 3, 16,19, 22, and 24. At the time of submission of the new SNPs, we noticed they were deposited by others and had an assigned SNP numbers of rs12964837, rs11872762, rs11877806, rs36124867, rs75363899, rs2853533, and rs72634355 respectively.
(TIF)

Figure S3 **Enlarged version of** **figure 1C** **(in main document).** The largest haplotype block spanning the *TYMS* gene and some parts in the 5′ UTR, including the VNTR and the mononucleotide repeats, and the 3′UTR, is expanded. Blocks 1 and 2 in this figure correspond to blocks 8 and 9 respectively, of Figure 1B (in main document). The unmatched marker 87 corresponding to SNP number rs3826626 was removed in this figure. The locations of the VNTR, the MR (mononucleotide repeats), and the 6-bp deletion/insertion polymorphism are given. The *TYMS* translational start codon is 13 bp downstream of the VNTR.
(TIF)

Table S1 **New SNPs identified in this study from the** ***TYMS*** **genetic region.**
(DOC)

Table S2 **A complete list of SNPs used for genotyping in this study.**
(DOC)
