# Supplementary material for: Analysis of Polymorphisms and Haplotype Structure of the Human Thymidylate Synthase Genetic Region: A Tool for Pharmacogenetic Studies
Source: PLoS One. 2012 Apr 5;7(4):e34426. doi: 10.1371/journal.pone.0034426 (PMC3320636; doi:10.1371/journal.pone.0034426)
Supplement: Figure S1 — Structure of the TYMS genetic region. The structure of the TYMS genetic region from coordinates 654,843–657,842 (UCSC genomic build GRCh37/hg19) is shown. The mononucleotide repeats (MR) and VNTR appear as bold underlined letters. The coordinates of the MR are reported in Table 2. The TYMS promoter is in italics. The primers used to amplify the MR are listed. (DOC) [file pone.0034426.s001.doc]

**Figure S1**

TTGCCATGACAATCGTAAACTGACATGGCACACTGGTGGGCACGTCTTAT

GGAAAACTACTTCTGCCCCATCCTTGTTTTGACTACTCCTCAATTTGGTC

CCATGTCAGAGCCCTGCCTCCAGAGTCAAGCCCTGTCTACCTCACAATTA

TTATGTATCAGGTTTTTAAAAAATAACACAAGTAAGGCTGGGCATGGTGG

CTCACGCCTTTAATCCCAGCACTTTGGGAGGCCGAGGTGGGCAGATCACT

TCAGATCAGAAGTCTGAGACAAGCCTGACCAACATGGTGAAACCCTGTCT

CTCCTAAAAATGC**AAAAAAAAAAAAAAAGAAAAAAAAA^1^**TTAGCTGGGCAT

GGTGGCAGGCGCCTATAGTTCCAGCTACTTGGGAGGCTGAGGCAGAATTG

CTTGAACCCATGCAGCGGAGTTTGCAGTGAGCTGAGCACCACTGCACTGC

AGCCTGAGGGACAGAGCAAGATTCCACCTC**AAAAAAAAAAAAAAAAAAAA**

**AAAA^2^**GGAAAAAGAATAACACAAGGAAATTAAACACAGATAACGTGCATCT

GGATAATCTGGAATATTTTCAGCCTGGGATCTTCCTCGTGCTCGGAAACG

TGTAGGGAAACGTGTAGGGACAGTTTTCAGCTGGTGTGGGGTGGGGGAAT

GCCAACATATGGTTGGCTTTCCACAGTCCTGCTAAAGCCCTGCAATGTGC

AGAACAAACCGGTCCAGCTAAGAAAAGTCCCACCCAAAATGCTGAGAGCA

CCTCTGTACTCAATCAACCGTTGAGGTAAAAGGATGCTTGCAAGTACAAC

ACATGTATCAGTGCATTTTAAACTCATTATAAAATCGTTCTGTAAACCAA

AAATAAAATTCTACGCCCAGAACCATCTGAAAAGACCACTCCTCTGGGTC

AAAGGCATTCCAAATTTAACCACAAAAACTAGTTCAGGCCATGATGGGAA

GGGGAGCCAGACATGCCTCATTATTCCCTCCTCCGTTTGGAATTTAGGCA

CAGCTGACCAGCATTGACATCAACACAGGCCTCAAGACAGGTAGAACACA

CTCTTTAAGTCTGACAAGAAACATTTACAATCTATCTCTCTAAAGCCTGC

TACCTGGAGGTTTAATCTGCATGATGAATCCATGGTCTCCACAACCCCTT

ATCTTAACCCAGATATTCCTTTCTATTCATTCAAATC**TTTTTTTTTTTTT**

**TCCCC^3^**ATACGGCTCTGTCACCCAGACTGGAGTGCAGTGGCGCTTTCTCAG

CTACTGCAAGCAACCTCCGCCTCCTGGGTTCAAGCGATTCTCCTACCTCA

GCCTCCTGAGTACCTGGGGTTACAGGCGTGTGCCACCTCGCCCCACTAAT

TTTTGTATTTTCAGTAGAGAGGGTTTCACTATGTTGGCCAGGATGGTCTC

AATCTCCTGACCTCGTGATCTGCCCGCCTCGACCTCCCAAGTGCTGGGAT

TACAGGCGTGAGCCACCGCGCCCAGCCCTCTATTGATTCCAGATCTTTAG

ATAACAATTCTTTCAACCAAATGTATAAAAAATCAAGTTGTAGCCCAACC

ACCTTGGGGACATGTTCTCAGGATCTGCTGAGGGCTCTATCACCGGCCAT

TGGTCACTCATATTTGGCTCAGAATAAATTTCTTCAGATATTTTACAGAG

TTTAACTCTTTTCATCAACAGTTCCCACTCCCCAGTGTTAATGTTATAAA

CAATTCAGTAAAACTGGGAAATGTGGTCTATTAAAAACTTGGGGCCGAGT

GCGGTGGCTCACGCCTGTAATCCCAGCACTTTGGGAGGCCAATGTGGGTG

GATCACCTGAGGTCAGGAGTTTGAGACCAGCCTGGCTAACATGGCGAAAC

CCCGTCTCTACTAAACATG**AAAAAAAAAAA^4^**TTAGCCGGGCGTGGTGGCGG

CGCCTGTAGTCCCAGCTACGCGAGAGGCTGAGGCAGCAGAATTGCTTGAA

CCCAGGAGGCGGAGGTTGCAGTGAGCCGAGATCGCACCATTGCACTCCAG

CCTGGGTGAGAGAGCGAGACTCTGTCTC**AAAAAAAAAAAAAAAA^5^**GACCGC

CAGGGCTCAAACAAAAAACCTCGGAAAAGCCCTGGCGGTC**TTTTTTTTTT**

**TTTTTTTTTTTTTTTT^6^**GGGACAGTCTTGCTCTGTCGCCCAGGCTGGAGTA

CAATGGTCGGATCTTGGCTCACTGCAACCTCTGCCTCCCAGGTTCAAGCA

ATTCTTCTGCCTCAGCCTCCCAAGTAGCCACCACGCCCAGCTAATTTTTG

TACTTTTAGTAGAGACGGGGGTTTCACCATGTTGTCCAGGCTGGTCTTGA

ACTCCTGACCTCAGGTGATCCACCCGCCTCGGCCCCCCAAAGTACTAGGA

TTACAGGCGTGAGCCACCGCGTCCAGCGCCCTGGCGGTTTTTAATCAAGT

AGAAAAGCTGCATTATACCACTTGCTTCGGTTGCTTCAGTGAGAACGAAG

AAATGGAAATGCAAATCCCTTATTAGTTGTAGGAAACAGATCTCAAACAG

CAGTTTTGTTGACAAGACCGCAGGAAAACGTGGGAAC***TGTGCTGCTGGCT***

***TAGAGAA*^7^**GGCGCGGTCGACCAGACGGTTCCCAAAGGGCGCAGTCCTTCCC

AGCCACCGCACCTGCATCCAGGTTCCCGGGTTTCCTAAGACTCTCAGCTG

TGGCCCTGGGCTCCGTTCTGTGCCACACCCGTGGCTCCTGCGTTTCCCCC

TGGCGCACGCTCTCTAGAGCGGGGGCCGCCGCGACCCCGCCGAGCAGGAA

GAGGCGGAGCGCGGGACGGCCGCGGGAAAAGGCGCGCGGAAGGGGTCCTG

**CCACCGCGCCACTTGGCCTGCCTCCGTCCCGCCGCGCCACTTGGCCTGCC**

**TCCGTCCCGCCGCGCCACTTCGCCTGCCTCCGTCCCC^8^**CGCCCGCCGCGCC

ATGCCTGTGGCCGGCTCGGAGCTGCCGCGCCGGCCCTTGCCCCCCGCCGC

ACAGGAGCGGGACGCCGAGCCGCGTCCGCCGCACGGGGAGCTGCAGTACC

^1^MR1

Forward- 5’FAM- CTCCAGAGTCAAGCCCTGTC

Reverse- ATGGGTTCAAGCAATTCTGC

^2^MR2

Forward- 5’FAM- CAGCTACTTGGGAGGCTGAG

Reverse- GCTGAAAACTGTCCCTACACG

^3^MR3

Forward- 5’FAM- CATCAACACAGGCCTCAAGA

Reverse- AAAAATTAGTGGGGCGAGGT

^4^MR4

Forward- 5’FAM- CATCAACACAGGCCTCAAGA

Reverse- AAAAATTAGTGGGGCGAGGT

^5^MR5

Forward- 5’FAM- GTAGTCCCAGCTACGCGAGAGGCTG

Reverse- AGGGCTTTTCCGAGGTTTTTTGTTTG

^6^MR6

Forward- 5’FAM- GCCAGGGCTCAAACAAAAAACCTCG

Reverse- AGGCTGAGGCAGAAGAATTGCTTGA

^7^*TYMS* promoter region

^8^ Three-repeat 28 bp VNTR (3R)
