# Supplementary figures and images for: Analysis of Polymorphisms and Haplotype Structure of the Human Thymidylate Synthase Genetic Region: A Tool for Pharmacogenetic Studies
Source: PLoS One. 2012 Apr 5;7(4):e34426. doi: 10.1371/journal.pone.0034426 (PMC3320636; doi:10.1371/journal.pone.0034426)

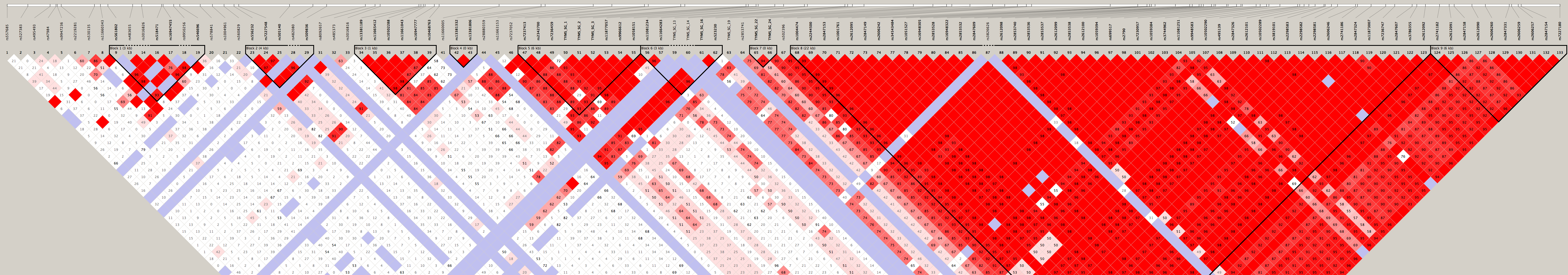

Supplement: Figure S2 — Enlarged version of figure 1B (in main document). Nine haplotype blocks (in triangular shape) were obtained by haplotype analysis using Haploview (see Materials and Methods), covering the 80 kb TYMS genetic region (depicted in Figure 1A). The reference SNP numbers (rs) are shown on top. The linkage disequilibrium (D′) is indicated in the small boxes colored red or blue as indicated by the color legend. The boxes in red or pink depict D′ to have very good correlation between SNPs. The boxes in blue or white indicate D′ to have poor correlation. Some newly discovered SNPs that were not in the public database at the time of analysis were named as TYMS_SG 1, 2, 3, 16,19, 22, and 24. At the time of submission of the new SNPs, we noticed they were deposited by others and had an assigned SNP numbers of rs12964837, rs11872762, rs11877806, rs36124867, rs75363899, rs2853533, and rs72634355 respectively. (TIF) [file pone.0034426.s002.tif]

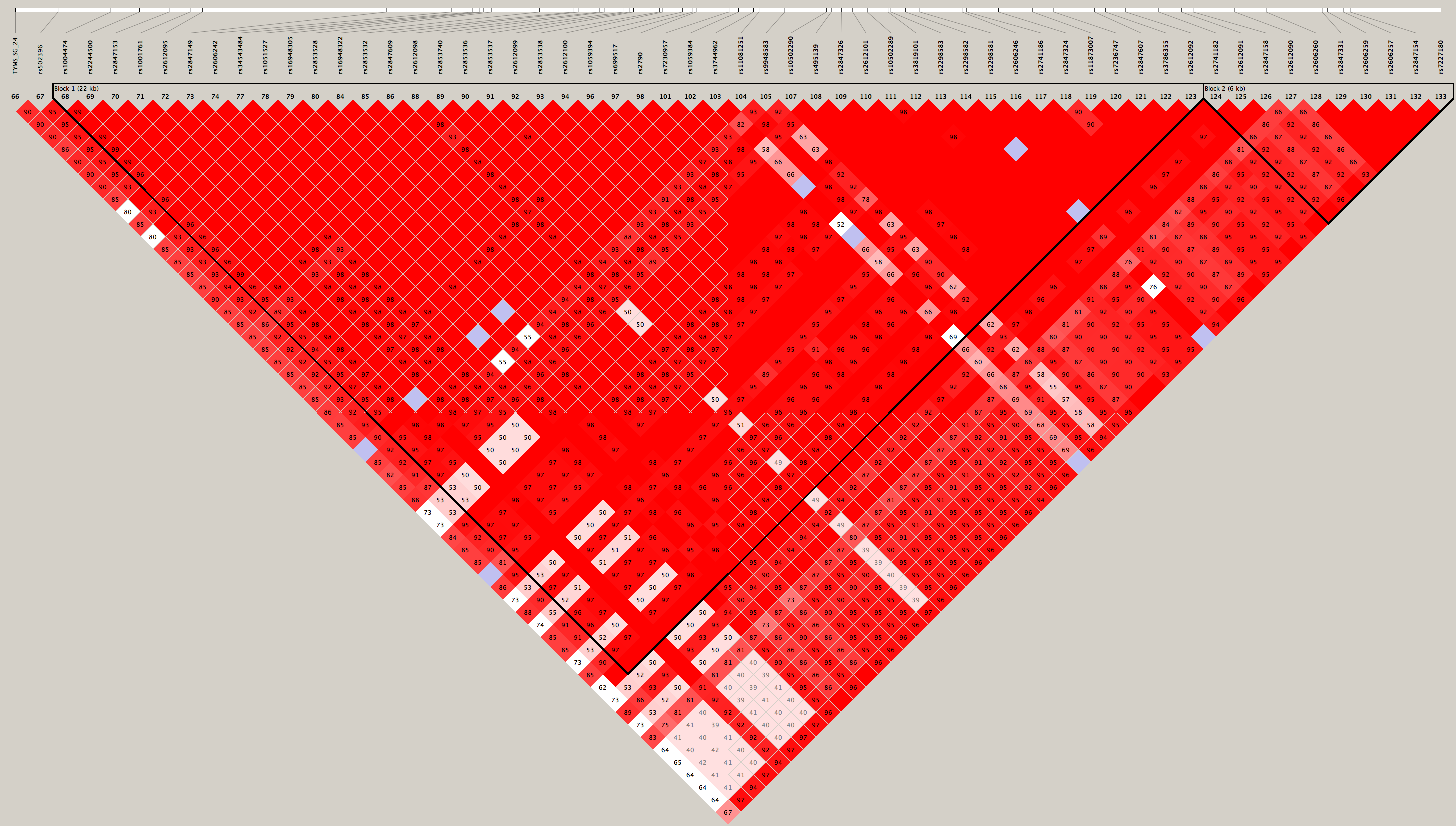

Supplement: Figure S3 — Enlarged version of figure 1C (in main document). The largest haplotype block spanning the TYMS gene and some parts in the 5′ UTR, including the VNTR and the mononucleotide repeats, and the 3′UTR, is expanded. Blocks 1 and 2 in this figure correspond to blocks 8 and 9 respectively, of Figure 1B (in main document). The unmatched marker 87 corresponding to SNP number rs3826626 was removed in this figure. The locations of the VNTR, the MR (mononucleotide repeats), and the 6-bp deletion/insertion polymorphism are given. The TYMS translational start codon is 13 bp downstream of the VNTR. (TIF) [file pone.0034426.s003.tif]
