# Supplementary material for: Analysis of Polymorphisms and Haplotype Structure of the Human Thymidylate Synthase Genetic Region: A Tool for Pharmacogenetic Studies
Source: PLoS One. 2012 Apr 5;7(4):e34426. doi: 10.1371/journal.pone.0034426 (PMC3320636; doi:10.1371/journal.pone.0034426)
Supplement: Table S1 — New SNPs identified in this study from the TYMS genetic region. (DOC) [file pone.0034426.s004.doc]

**Table S1.** New SNPs from the *TYMS* genetic region

| **Name** | **Coordinate^1^** | | **SNP** |
| --- | --- | --- | --- |
| TYMS_SG_11 | 641987 | A/C | |
| TYMS_SG_13 | 642875 | C/T | |
| TYMS_SG_14 | 643015 | G/A | |

^1^Genomic positions according to NCBI genome build 36.1
