# Supplementary material for: Analysis of Polymorphisms and Haplotype Structure of the Human Thymidylate Synthase Genetic Region: A Tool for Pharmacogenetic Studies
Source: PLoS One. 2012 Apr 5;7(4):e34426. doi: 10.1371/journal.pone.0034426 (PMC3320636; doi:10.1371/journal.pone.0034426)
Supplement: Table S2 — A complete list of SNPs used for genotyping in this study. (DOC) [file pone.0034426.s005.doc]

**Table S2.** Complete list of SNPs included in this study.

| **Serial No.** | **Locus name** | **Coordinate^1^** | **SNP** |
| --- | --- | --- | --- |
| 1 | rs557685 | 600272 | T/C |
| 2 | rs527383 | 601322 | A/G |
| 3 | rs495493 | 602453 | A/G |
| 4 | rs4798418 | 602752 | T/C |
| 5 | rs9947236 | 602818 | T/C |
| 6 | rs2219281 | 603019 | T/G |
| 7 | rs530135 | 604707 | A/G |
| 8 | rs11660343 | 605013 | A/C |
| 9 | rs561802 | 605393 | T/C |
| 10 | rs16947400 | 605649 | T/C |
| 11 | rs16947413 | 606194 | T/C |
| 12 | rs480759 | 606316 | A/G |
| 13 | rs483655 | 606652 | T/C |
| 14 | rs3016826 | 607109 | A/G |
| 15 | rs9960753 | 607927 | A/T |
| 16 | rs518471 | 608123 | T/C |
| 17 | rs16947455 | 608200 | T/C |
| 18 | rs9950256 | 608740 | C/G |
| 19 | rs546696 | 609049 | A/C |
| 20 | rs578841 | 610088 | T/C |
| 21 | rs1004961 | 610708 | A/C |
| 22 | rs560829 | 611299 | C/G |
| 23 | rs556232 | 611807 | A/G |
| 24 | rs7227548 | 612047 | C/G |
| 25 | rs505140 | 614630 | T/G |
| 26 | rs495799 | 614823 | T/C |
| 27 | rs7239762 | 615144 | T/C |
| 28 | rs482080 | 615819 | A/G |
| 29 | rs509836 | 616517 | A/C |
| 30 | rs540094 | 617628 | A/G |
| 31 | rs8092657 | 618486 | A/C |
| 32 | rs485373 | 618979 | T/C |
| 33 | rs3016816 | 619238 | C/G |
| 34 | rs13381189 | 621210 | T/G |
| 35 | rs11665412 | 621313 | T/C |
| 36 | rs10502288 | 621434 | A/G |
| 37 | rs11661043 | 621525 | T/C |
| 38 | rs16947777 | 621993 | A/G |
| 39 | rs10468763 | 622238 | T/C |
| 40 | rs16947823 | 624558 | A/C |
| 41 | rs11660005 | 625287 | A/G |
| 42 | rs13381332 | 627423 | C/G |
| 43 | rs13381806 | 627630 | T/C |
| 44 | rs28880359 | 630075 | T/C |
| 45 | rs11663153 | 631023 | A/C |
| 46 | rs7237052 | 632677 | A/C |
| 47 | rs7237413 | 632875 | T/C |
| 48 | rs2342700 | 634526 | C/G |
| 49 | rs7236459 | 634733 | A/G |
| 50 | rs12964837 | 637846 | T/C |
| 51 | rs11872762 | 637961 | C/G |
| 52 | rs11877806 | 638356 | T/C |
| 53 | rs11877057 | 638464 | A/G |
| 54 | rs9966612 | 639310 | A/G |
| 55 | rs1058151 | 639758 | T/C |
| 56 | rs11081234 | 640815 | A/G |
| 57 | rs11664283 | 640967 | A/G |
| 58 | TYMS_SG_11 | 641987 | A/C |
| 59 | TYMS_SG_13 | 642875 | T/C |
| 60 | TYMS_SG_14 | 643015 | A/G |
| 61 | rs36124867 | 643256 | A/C |
| 62 | rs523230 | 643846 | A/G |
| 63 | rs75363899 | 646356 | T/C |
| 64 | rs2853741 | 647351 | T/C |
| 65 | rs2853533 | 648044 | C/G |
| 66 | rs72634355 | 648312 | T/C |
| 67 | rs502396 | 649235 | T/C |
| 68 | rs1004474 | 650382 | A/G |
| 69 | rs2244500 | 651004 | T/C |
| 70 | rs2847153 | 651646 | A/G |
| 71 | rs1001761 | 652102 | T/C |
| 72 | rs2612095 | 652369 | T/C |
| 73 | rs2847149 | 656370 | A/G |
| 74 | rs2606242 | 657769 | C/G |
| 75 | rs35626549 | 657869 | T/C |
| 76 | rs571377 | 658108 | A/G |
| 77 | rs34543484 | 658238 |  |
| 78 | rs1051527 | 658375 | C/G |
| 79 | rs16948305 | 658464 | T/C |
| 80 | rs2853528 | 658649 | T/C |
| 81 | rs12326481 | 658766 | T/C |
| 82 | rs596909 | 659086 | T/G |
| 83 | rs35710611 | 659186 | T/C |
| 84 | rs16948322 | 659683 | T/C |
| 85 | rs2853532 | 660413 | T/C |
| 86 | rs2847609 | 660540 | A/G |
| 87 | rs3826626 | 660887 | T/C |
| 88 | rs2612098 | 660994 | A/C |
| 89 | rs2853740 | 661104 | T/C |
| 90 | rs2853536 | 661519 | T/C |
| 91 | rs2853537 | 661647 | A/T |
| 92 | rs2612099 | 661726 | A/G |
| 93 | rs2853538 | 662287 | T/C |
| 94 | rs2612100 | 662362 | A/G |
| 95 | rs9953447 | 662695 | A/G |
| 96 | rs1059394 | 662791 | T/C |
| 97 | rs699517 | 663015 | T/C |
| 98 | rs2790 | 663085 | A/G |
| 99 | rs16948409 | 663210 | T/G |
| 100 | rs16948421 | 663394 | A/G |
| 101 | rs7230957 | 663788 | A/G |
| 102 | rs1059384 | 663994 | T/C |
| 103 | rs3744962 | 664319 | A/G |
| 104 | rs11081251 | 664439 | A/C |
| 105 | rs9948583 | 664999 | T/C |
| 106 | rs8089075 | 665271 | T/C |
| 107 | rs10502290 | 665902 | T/C |
| 108 | rs495139 | 666007 | C/G |
| 109 | rs2847326 | 666227 | A/T |
| 110 | rs2612101 | 666472 | T/C |
| 111 | rs10502289 | 666788 | A/T |
| 112 | rs3819101 | 667239 | A/G |
| 113 | rs2298583 | 667301 | A/G |
| 114 | rs2298582 | 667620 | T/G |
| 115 | rs2298581 | 667930 | C/G |
| 116 | rs2606246 | 668846 | T/C |
| 117 | rs2741186 | 668946 | A/G |
| 118 | rs2847324 | 669636 | T/C |
| 119 | rs11873007 | 670379 | T/C |
| 120 | rs7236747 | 670837 | A/G |
| 121 | rs2847607 | 671723 | T/C |
| 122 | rs3786355 | 671961 | A/G |
| 123 | rs2612092 | 672398 | T/C |
| 124 | rs2741182 | 673115 | C/G |
| 125 | rs2612091 | 673606 | T/C |
| 126 | rs2847158 | 673860 | T/C |
| 127 | rs2612090 | 674765 | T/C |
| 128 | rs2606260 | 675446 | A/G |
| 129 | rs2847331 | 676661 | T/C |
| 130 | rs2606259 | 676781 | T/C |
| 131 | rs2606257 | 677118 | T/G |
| 132 | rs2847154 | 677269 | A/G |
| 133 | rs7227180 | 679241 | C/G |

^1^Genomic positions according to NCBI genome build 36.1
